# Adaptation of a visualized loop-mediated isothermal amplification technique for field detection of *Plasmodium vivax *infection

**DOI:** 10.1186/1756-3305-4-115

**Published:** 2011-06-21

**Authors:** Zhi-Yong Tao, Hua-Yun Zhou, Hui Xia, Sui Xu, Han-Wu Zhu, Richard L Culleton, Eun-Taek Han, Feng Lu, Qiang Fang, Ya-Ping Gu, Yao-Bao Liu, Guo-Ding Zhu, Wei-Ming Wang, Ju-Lin Li, Jun Cao, Qi Gao

**Affiliations:** 1 Department of Parasitology, Medical College of Soochow University, Suzhou 215123, People's Republic of China; 2 Jiangsu Institute of Parasitic Diseases, Key Laboratory on Technology for Parasitic Disease Prevention and Control, Ministry of Health, Meiyuan Yangxiang 117, Wuxi 214064, People's Republic of China; 3 Department of Parasitology, Bengbu Medical College, 2600 Donghai Dadao Road, Bengbu 233030, People's Republic of China; 4 Malaria Unit, Institute of Tropical Medicine, Nagasaki University, 1-12-4 Sakamoto, Nagasaki 852-8523, Japan; 5 Department of Parasitology, Kangwon National University College of Medicine, Chuncheon, Republic of Korea; 6 Chenzhou Center for Disease Control and Prevention, Chenzhou, 423000, People's Republic of China

## Abstract

**Background:**

Loop-mediated isothermal amplification (LAMP) is a high performance method for detecting DNA and holds promise for use in the molecular detection of infectious pathogens, including *Plasmodium *spp. However, in most malaria-endemic areas, which are often resource-limited, current LAMP methods are not feasible for diagnosis due to difficulties in accurately interpreting results with problems of sensitive visualization of amplified products, and the risk of contamination resulting from the high quantity of amplified DNA produced. In this study, we establish a novel visualized LAMP method in a closed-tube system, and validate it for the diagnosis of malaria under simulated field conditions.

**Methods:**

A visualized LAMP method was established by the addition of a microcrystalline wax-dye capsule containing the highly sensitive DNA fluorescence dye SYBR Green I to a normal LAMP reaction prior to the initiation of the reaction. A total of 89 blood samples were collected on filter paper and processed using a simple boiling method for DNA extraction, and then tested by the visualized LAMP method for *Plasmodium vivax *infection.

**Results:**

The wax capsule remained intact during isothermal amplification, and released the DNA dye to the reaction mixture only when the temperature was raised to the melting point following amplification. Soon after cooling down, the solidified wax sealed the reaction mix at the bottom of the tube, thus minimizing the risk of aerosol contamination. Compared to microscopy, the sensitivity and specificity of LAMP were 98.3% (95% confidence interval (CI): 91.1-99.7%) and 100% (95% CI: 88.3-100%), and were in close agreement with a nested polymerase chain reaction method.

**Conclusions:**

This novel, cheap and quick visualized LAMP method is feasible for malaria diagnosis in resource-limited field settings.

## Background

Between the years 2000 and 2008, there was re-emergence of *Plasmodium vivax *malaria in the central part of the People's Republic of China (P. R. China) [1]. Rapid and accurate diagnosis of malaria is not only crucial for patient treatment, but also important for disease control, especially during attempts at elimination, as *P. vivax *infections are often found at low parasite densities, and any missed cases of malaria could be a potential source of local transmission. Microscopic examination of blood films is the most wildly used diagnostic approach in the field and still remains the 'gold' standard. However, this method is labour-intensive, requires well-trained experts and may result in therapeutic delays. Recently developed lateral flow-based malaria rapid diagnostic tests (RDTs) have proved useful in *P. falciparum*-endemic countries, as the sensitivity of RDTs against *P. falciparum *histidine-rich protein II (PfHRP-II) and *P. falciparum *lactate dehydrogenase (PfLDH) is high [2,3]. In contrast, RDTs for *P. vivax *are currently not as sensitive as those for *P. falciparum*, due to the low parasitaemia and lack of abundantly expressed specific antigens [3].

Loop-mediated isothermal amplification (LAMP) is a high performance method for detecting DNA [4], which holds promise for use in molecular diagnosis of pathogens in the first line battle against infectious diseases, including malaria and tuberculosis [5-8]. LAMP uses a set of primers that initiate large scale nucleic acid synthesis by *Bst *DNA polymerase at isothermal conditions. As a candidate method for field malaria diagnosis, a few different primer sets targeting numerous genes have previously been developed. It has been claimed that the LAMP method can detect as few as 100 copies of DNA template in blood samples (equal roughly to 5 parasites/μl) [9,10]. This sensitivity is notably higher than any known immunochromatography-based RDTs, which are recommended by WHO as part of the global malaria control strategy [11].

LAMP results can be accurately observed using a real-time turbidimeter [12,13], but in light of the fact that most malaria cases occur in poor and rural regions, a less expensive and simplified method should be considered for maximum impact. Although introducing manganese into the reaction can generate visible white precipitation [6], such turbidity is usually too weak to be observed by the naked eye, and bias can occur due to inter-observer differences. Calcein can be added to the reaction mixture to visualize the LAMP result, but the colour change induced is less noticeable than that obtained with the routine nucleic acid dye SYBR Green I [14], which, unfortunately, inhibits the amplification reaction, so cannot be added prior to the reaction. Thus, a fluorescence dye is often added after the reaction has occurred in order to stain LAMP products [15]. However, this involves opening the tubes in which the reaction has occurred prior to visualization, and this procedure holds the risk of contamination due to the high copy number of amplified LAMP products [16]; even a small amount of amplicon aerosol released to the laboratory environment could cause contamination that is very difficult to eliminate. This kind of contamination risk is an obstruction to the use of the LAMP method as a field diagnostic tool for malaria and other infectious diseases [15].

Hot-start polymerase chain reaction (PCR) had, historically, been dependent on the use of wax beads to separate the polymerase from the rest of the reaction mix prior to an initial heating step [17]. Unlike the high-low-medium temperature cycles of general PCR, LAMP uses a constant temperature of approximately 65°C for amplification. This allows the addition of a solid bead of wax containing dye to the LAMP reaction mix prior to amplification. The wax will remain unmelted during amplification and can then be exposed to a higher temperature following the cessation of the reaction, allowing the dye to be released and the products visualized. Microcrystalline wax has a higher molecular weight and melting point than paraffin waxes, and is also more elastic and easy to cast into a mold.

In this study, we chose microcrystalline wax and SYBR Green I as the basic materials in order to develop a wax-dye capsule for the establishment of a novel visualized LAMP detection assay for *P. vivax*, which avoids the necessity of opening the reaction tube following amplification, and determined its feasibility as a RDT for *P. vivax *malaria field diagnosis in a resource-limited setting.

## Methods

### Preparation of microcrystalline wax-dye capsule

**Figure 1:**
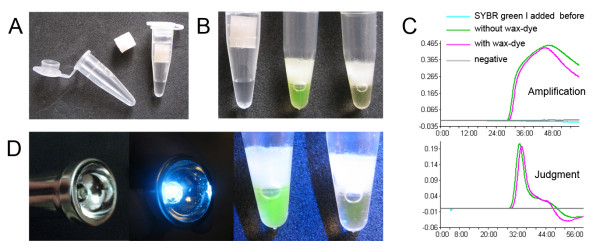
**Using microcrystalline wax-dye capsule in LAMP detection**. (A) Dye contained within a microcrystalline wax capsule; there is no contact between the wax and the LAMP mixture before amplification. (B) After isothermal amplification the capsule remains intact, and the dye was released by melting at 95°C; positive tube shows bright green, negative is orange, while cooling wax turns into solid barrier. (C) The LAMP reaction was monitored by a real-time turbidimeter, amplification and judgment curves of tubes with capsule added are similar to those without capsules, and the addition of SYBR Green I directly to the reaction prior to amplification completely inhibited the reaction. (D) A modified household flashlight with one 475 nm LED, and the result of blue light excitation.

To create the wax-dye capsule, the distal end of 1 ml disposable syringe was removed, and the remaining stem part and plug used as a mold. A small amount of microcrystalline wax (no. 85#, melting point = 85°C; Sinopec, Nanyang, P. R. China) was placed in a stainless steel lab spoon and heated using the flame of a spirit lamp to melt the wax. 0.3 ml of liquid wax was then poured into the syringe mold and was cooled so that the wax solidified. Then, 1 μl of SYBR Green I (1,000 X) solution was added to the wax surface in the syringe mold. Subsequently, another 0.3 ml of liquid wax was poured on top of the dye solution. When the final wax layer cooled, a solid capsule was created and pushed out of the mold using the plug. Such capsules can be prepared in batches and stored at -20°C until use (Figure 1A).

### Field sample collection and DNA extraction

A total of 89 dried blood spot (DBS) samples were collected in the northern part of Anhui province, P. R. China, where *P. vivax *is the only malaria parasite transmitted. Samples were collected from febrile patients with suspected malaria infections between July 2008 and August 2009. Following the acquisition of written consent, blood was draw into a heparin treated vacuum tube, 70 μl of whole blood was spotted onto filter paper (Whatman, S & S 903), air dried and stored at -20°C. Simultaneously, blood films were made and stained by Giemsa's solution for microscopy examination, and all slides were read by two expert microscopists, each with more than 10 years of experience. A rapid DNA extraction method for DBS was modified from the method previously described by Bereczky *et al. *[18]. In brief, a spot of DBS filter paper was cut into four equal pieces, one piece (~18 μl whole blood) placed into a 0.5 ml centrifuge tube, 100 μl of double distilled water added, left to stand for 5 min, and then boiled for 10 min at 100°C. The supernatant, containing DNA, was subsequently used in both visualized LAMP and nested PCR analysis.

### LAMP primers design

**Figure 2:**
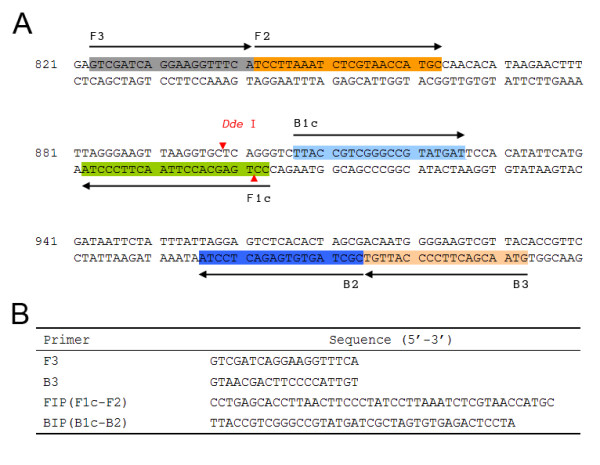
**Location and sequence of LAMP primer for *Plasmodium vivax *detection**. (A) Partial sequence of *P. vivax *mtDNA and the location of four primers: F3, B3, FIP (F1c-F2) and BIP (B1c-B2), arrows indicate the direction of extension, and a restriction enzyme *Dde *I site located in the F1c region. (B) Sequence of primers for *P. vivax *mtDNA LAMP reaction.

The *P. vivax *mitochondrial DNA (mtDNA) was chosen as the target region, because it is a plasmid-like multicopy non-nuclear DNA and is easy to extract using the boiling method. The full length of *P. vivax *mtDNA (GenBank accession no. AY598140.1) is 5,990 bp with an overall GC content of 30.5%. A relative high GC-rich (44%) 565 bp fragment (position 694 to 1258) of *P. vivax *mtDNA was selected as target sequence for primer design and LAMP detection. Primer design was performed using the online tool Primer Explorer version 4.0 http://primerexplorer.jp/elamp4.0.0/index.html. All parameters were set by default, whereas a set of primers were chosen and synthesized (Figure 2).

### LAMP conditions and visualization

The preparation of the LAMP reaction solutions was carried out according to Zhu *et al. *[19]; each 25 μl reaction mixture contained the following: 20 mM Tris-HCl pH 8.8, 10 mM KCl, 10 mM (NH_4_)_2_SO_4_, 0.1% Tween 20, 8 mM MgSO_4_, 1.4 mM each of dNTPs, 1.6 μM FIP, 1.6 μM BIP, 0.2 μM F3, 0.2 μM B3, 8 U *Bst *DNA polymerase. 1 μl of DNA template was added to the reaction tube and briefly vortexed, before being placed into a real-time turbidimeter (LA 320c, Teramecs; Kyoto, Japan), and one wax-dye capsule placed into the tube before sealing with a cap. LAMP reaction conditions were: 65°C for 60 min, during amplification, the lid temperature was maintained at 80°C, and the positive threshold of turbidity was set to 0.1, reaction was terminated at 80°C for 5 min. Following amplification, reaction tubes were transferred to a general PCR machine (Mastercycler, Eppendorf; Hamburg, Germany), the temperature of heat block and lid were adjusted to 95°C for 5 min to melt the wax-dye capsule and to release SYBR Green I into the reaction mixture. Then the block was cooled to 4°C for 5 min. Reaction tubes were removed and observed by the naked eye (Figure 1B). For field DBS samples, all LAMP reactions were carried out in a water bath: 65°C for 60 min, 95°C for 5 min at room temperature for 10 min. A simple model tool was developed for blue light excitation of SYBR Green I. One 475 nm (visible blue light) LED element was used to replace the bulb of a household flashlight (Figure 1D). Then LAMP results were observed and determined under this blue light.

### Construction of positive control plasmid DNA

PCR products obtained following amplification of DNA with LAMP primers F3 and B3c were inserted in to pGEM-T vector (Promega). Each 20 μl PCR mixture contained: 10 × PCR buffer 2 μl, 25 mM MgCl_2 _1.2 μl, 2.5 mM each dNTPs 1.6 μl, *Taq *polymerase 0.1 μl, DNA template 1 μl, distilled deionized water (DDW) 13.2 μl, 10 μM F3 primer 1.0 μl and 10 μM B3c primer 1.0 μl. The PCR was performed at 95°C for 5 min for denaturation, followed by 35 cycles; 95°C for 30 s, 56°C for 30 s, 72°C for 30 s, and final extension 72°C for 5 min. PCR products were cloned into vector following a standard protocol [20]. The recombinant pGEM-T plasmid DNA cloned *P. vivax *mtDNA fragment (pGEM-PvMito) was extracted by a Miniprep kit (Sangon) from *Escherichia coli *(DH5α strain), the concentration was measured by a spectrophotometer and copy numbers were calculated.

### Analytical sensitivity and specificity of LAMP

Following the establishment of the visualized closed tube LAMP method for *P. vivax *mtDNA detection, LAMP products were digested by *Dde *I to assess the specificity of amplification. Subsequently, sensitivity was assessed in the following manner; recombinant pGEM-PvMito plasmid DNA was serially diluted (10-fold), so that each sample contained 1.0 × 10^6 ^to 1.0 × 10^2 ^copies, and LAMP reactions were carried out as above.

A DBS DNA sample from one patient with parasitaemia of 11,000 parasites/μl of blood was 10-fold serially diluted by using either sterile water or mixed negative DBS DNA sample respectively. The *P. vivax *DNA serial dilution rate is from 1 to 10,000. LAMP detection was carried out as above, and compared with nested PCR.

### Nested PCR

A well established nested PCR targeting the 18S rRNA gene was performed as described previously with optimization for the detection of *P. vivax *[21]. Briefly, for the first round, 1 μl DBS DNA sample was used as DNA template, and primers were: rPLU6: 5'-TTAAAATTGTTGCAGTTAAAACG-3' and rPLU5: 5'-CCTGTTGTTGCCTTAAACTTC-3'; and the product of amplification for *P. vivax *is about 1.2 kb. DNA amplification as carried out under the following conditions: 95°C for 5 min, then followed by 24 cycles at 95°C for 30 s, 56°C for 30 s, 72°C for 80 s, and final extension at 72°C for 10 min. For the second round, 1 μl of PCR product of first round was used as template, and primers were: rVIV1: 5'-CGCTTCTAGCTTAATCCACATAACTGATAC-3' and rVIV2: 5'-ACTTCCAAGCCGAAGCAAAGAAAGTCCTTA-3'; the product being about 120 bp. The amplification conditions were: 95°C for 5 min, followed by 30 cycles at 95°C for 30 s, 56°C for 30 s, 72°C for 30 s, and final extension at 72°C for 5 min. PCR products were analysed by running for 15 min on a 2.0% agarose gel stained with ethidium bromide at 100 v.

### Statistical analysis

The sensitivity and specificity of novel visualized LAMP and nested PCR diagnosis were compared to microscopy (considered as 'gold' standard), and 95% confidence intervals (CI) and p values were calculated using SAS software (SAS Institute; Cary, NC, USA).

### Ethical statement

The study was approved by the institutional review board (IRB00004221) of Jiangsu Institute of Parasitic Diseases, Wuxi, P. R. China. Questionnaire surveys, physical examination and laboratory work were conducted after the purpose and procedure of the study had been explained to participants, who were given the right to withdraw from the study at any time. Written consent form was obtained from each participant or his/her guardian.

## Results

### Establishment a closed-tube visualization LAMP system

Standard thin wall PCR tubes were used, and 25 μl LAMP reaction solutions were placed in the bottom section. The fluid level constantly remained below the interface of the tapered bottom section and the straight column body section. This enabled space for insertion of the column-like wax-dye capsule without contacting the reaction solution (Figure 1A). Following LAMP amplification at 65°C for 60 min, reaction tubes were removed from the water bath, and all wax capsules in the tubes were observed to have remained intact during the reaction process. All tubes were then heated at 95°C for 5 min, melting the wax capsules. Subsequently, tubes were allowed to cool to room temperature, and a thick white solid layer was observed to have formed above the reaction solution (Figure 1B).

Positive tubes displayed a visible green colour, whereas the negative tubes maintained the orange colour of unbound SYBR Green I. When visible blue l or UV light was used to excite the SYBR Green I, positive tubes showed a higher degree of green fluorescence, and the negative tubes remained orange. However, without using the capsule, when same amount of SYBR Green I was added to the LAMP tube before the reaction, the amplification was completely inhibited (Figure 1C).

### Sensitivity and specificity of visualized LAMP method

**Figure 3:**
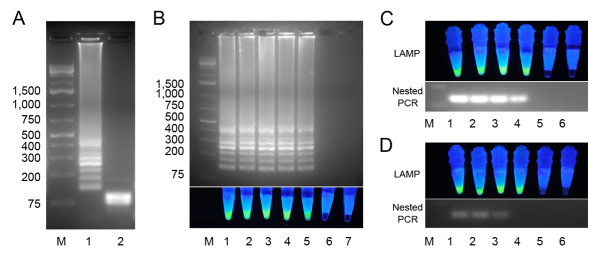
**Detection limit of *Plasmodium vivax *mtDNA LAMP and nested PCR**. (A) Agarose gel electrophoresis of LAMP product before (1) and after (2) digestion by *Dde *I (Fermentas). M: DNA ladder (1 kb Plus, Fermentas). (B) pGEM-PvMito plasmid was 10-fold serially diluted and tested by LAMP, M: DNA ladder, 1: 1.0 × 10^6^, 2: 1.0 × 10^5^, 3: 1.0 × 10^4^, 4: 1.0 × 10^3^, 5: 1.0 × 10^2^, 6: 1.0 × 10^1^, 7: DDW, results were observed by both electrophoresis and under blue light. (C) A *P. vivax *DBS sample with parasitaemia at 11,000 parasites/μl blood was serially diluted with DDW and tested by LAMP and nested PCR: M: DNA ladder, 1, undiluted, 2: 1:10, 3, 1:100, 4, 1:1,000, 5, 1:10,000; 6, DDW. (D) The same *P. vivax *DBS sample of (C) was diluted using mixed negative DBS sample and was tested as (C).

Amplified LAMP products were analysed using *Dde *I restriction enzyme, bands of ~80 bp were observed (Figure 3A), which were in accordance with theoretical analysis. pGEM-PvMito plasmids were 10-fold serially diluted in order to determine the detection limit of the visualized *P. vivax *mtDNA LAMP method. Electrophoresis results revealed that it was possible to detect 1.0 × 10^2 ^copies (Figure 3B). A DBS DNA sample from one *P. vivax *patient with parasite density of 11,000 parasites/μl of blood was serial diluted by DDW or mixed with negative DBS DNA samples, visualized LAMP method could detect both samples series at a 1:1,000 dilution rate (Figure 3C). The nested PCR was also able to amplify DNA at this concentration when the sample was diluted with DDW. However, the sensitivity of the nested PCR was reduced to 1:100 dilution, when the dilution agent was negative DBS DNA extraction supernatant (Figure 3D).

### Field sample test using visualized LAMP

**Figure 4:**
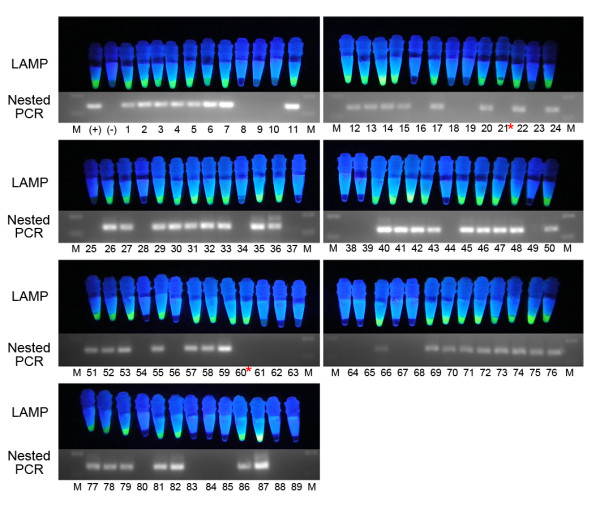
**Results of LAMP and nested PCR**. A total of 89 DBS DNA samples were tested by visualized LAMP method and nested PCR. LAMP positive tubes show bright green fluorescence, negative tubes remain orange. Nested PCR product for *P. vivax *18S rRNA gene is about 120 bp. (+): positive control, (-): negative control, 1-89: DBS DNA samples. * Those 2 DBS samples were positive for LAMP but negative for nested PCR method.

A total of 89 DBS DNA samples were tested by visualized LAMP and nested PCR. Giemsa-stained thick and thin blood films were examined by microscopy for comparison; 60 of the 89 samples were positive by microscopy. For visualized LAMP method, 59 of 89 were positive, for nested PCR, 57 of 89 were positive. Two DBS samples were positive for LAMP but negative for the nested PCR method. Compared to the referential microscopy method, the sensitivity of LAMP and nested PCR were 98.3% (95% CI: 91.1-99.7%) and 95.0% (95% CI: 86.3-98.3%), respectively. The specificity was 100% for both methods (Table 1 and Figure 4). Compared to microscopy, there were no significant differences observed (P < 0.05).

**Table 1:** Results of malaria detection by visualized LAMP, nested PCR, and microscopy

Comparison	No. of samples	
	
	Microscopy positive	Microscopy negative
LAMP *vs*. microscopy (*n *= 89)		
LAMP positive	59	0
LAMP negative	1	29
Nested PCR *vs*. microscopy (*n *= 89)		
Nested PCR positive	57	0
Nested PCR negative	3	29

## Discussion

Use of LAMP to detect pathogens has many advantages, especially with regard to its high sensitivity and specificity and the fact that it requires only small amounts of target DNA to be present in a sample for positive diagnosis. It may also be considered an attractive method for use in resource-limited areas, due to its low cost, and the minimal requirements for specialist equipment or expertise. However, according to Wastling *et al. *[22], visualization *via *observation of the colour change of calcein-MnCl_2 _methodology is less sensitive. SYBR Green I is one of the most sensitive general nuclei acid fluorescence dyes available [23]. Compared to calcein, which is now often used as a fluorescent dye in closed-tube LAMP analyses, SYBR Green I can be excited by both UV and visible light. The colour of this dye changes from orange to bright green fluorescence when conjugated into DNA and excited by UV or blue light, and this change is more dramatic than that achieved with calcein [14]. Furthermore, blue light excitation can be easily established without using UV light, which can be expensive and hazardous. In this report, we made a simple handset to provide a blue light source to excite SYBR Green I, the use of which resulted in easier discrimination between positive and negative reactions. The handset is based on a household single LED flashlight with the bulb changed to a 475 nm LED. This thumb size portable tool is reusable and the total cost is below US$ 0.5.

Even though SYBR Green I is advantageous due to its high sensitivity in the detection of amplified nuclei acids, reaction inhibition occurs if the dye is directly added to LAMP reaction solutions at concentrations required for visualization. In our closed-tube LAMP system, a microcrystalline wax capsule was used as a dye container, and effectively prevented this inhibitive effect by keeping the dye separate from the reaction mixture until the reaction had ceased. We tested this method using a real-time turbidimeter, and found that there was no significant change in the amplification curve between tubes with and without wax-dye. This result suggests that no interferential material was released by the wax-dye capsule during isothermal amplification at 65°C. In contrast, without using the capsule, the reaction was completely inhibited by SYBR Green I. This novel method also has the additional advantage of reducing the risk of aerosol contamination of LAMP amplicons. During the high temperature heating process, the wax shell melts into a low density liquid, which floats on top of the fluid level. The dye core sinks into reaction solution to form a mixture. After cooling down, the wax solidifies, and forms a barrier that seals the amplification solution into the bottom of tube. The contamination risk was therefore reduced during and after testing.

DNA extraction procedure is one of the critical steps for PCR-based field molecular diagnostic tools, as factors in human blood may inhibit PCR reactions. The rapid boiling method for extracting DNA from DBS is simple and fast, and suitable for use in the field. With a washing step, it can provide relatively high quality template DNA for PCR, but this step prolongs the turnaround time. Compared to *Taq *polymerase, LAMP uses *Bst *enzyme, which is not affected by blood proteins [24]. In this study, one DBS DNA sample from a *P. vivax *patient with parasite density of 11,000 parasites/μl was serially diluted with DDW or mixed negative DBS DNA solution, and subjected to both LAMP and nested PCR. There was no difference between the nested PCR and LAMP sensitivities when the diluent was DDW, but the sensitivity of the nested PCR was 10 times lower than LAMP when template was diluted with supernatant from the DNA extraction process carried out on parasite-negative blood samples. This result suggests that *Taq *polymerase-based nested PCR is more vulnerable than LAMP to interferential substances in DBS DNA extract. However, it should be noted that the target of the LAMP assay is the mitochondrial genome, which is known to have many copies per parasite (probably 20-50 times more) than the nuclear genome, on which the 18S rRNA gene targeted by the nested PCR is located.

A total of 89 field DBS samples were subjected to the rapid boiling DNA extraction method, and were analysed by visualized LAMP and nested PCR. The sensitivity and specificity of the LAMP assay were similar to previous reports [9,25,26], and the sensitivity of LAMP is slightly higher than nested PCR in this study, which may have been caused by inhibiting factors present in extracted DNA, or the copy number difference of the target genes. Nested PCR is based on 18S rRNA gene, the copy number of which is about 4-8 per *Plasmodium *spp. genome [27], whereas LAMP is based on mtDNA, which, for one *Plasmodium *parasite, there are about 30-150 copies [28]. According to Guo *et al. *[29], the highest yield method for extracting mtDNA for diagnosis purpose is phenol-chloroform-isoamyl alcohol, as using silica-based column methods may result in mtDNA lose. The rapid boiling method is both fast and low cost, making it suitable for mtDNA-based molecular diagnostic methods.

## Conclusions

A visualized closed-tube LAMP method was established utilising microcrystalline wax-dye capsules, which can deliver highly sensitive DNA fluorescence dye to the reaction mixture following amplification by melting the wax shell through heating to 95°C. This modified visualized LAMP method retains high sensitivity and specificity even using the rapid boiling DNA extraction from DBS technique. Furthermore, in order to assess its suitability for use in the field, a total of 89 field samples were tested by this method. Compared to microscopy, considered as the 'gold' standard of malaria diagnosis, very high sensitivity and specificity of this modified visualized LAMP method were found, and were in good agreement with a classical *P. vivax *nested PCR method. This novel, cheap and quick visualized LAMP method holds promise for malaria diagnosis in resource-limited settings. The procedure offers great potential and merits further and comprehensive field validation before it can become part of routine surveillance-response approaches in China or elsewhere.

## Competing interests

The authors declare that they have no competing interests.

## Authors' contributions

ZYT, JC and QG conceived the study and participated in its design and coordination. ZYT, SX and HWZ carried out LAMP analyses. HYZ, HX, FL, FQ, YPG, YBL, GDZ, WMW and JLL contributed expertise in field sample collection, microscopy and molecular analyses. ZYT wrote the manuscript. JC, RLC and ETH assisted with the interpretation of the results and manuscript drafting. All authors read and approved the final manuscript.
